# NcRNAs in Vascular and Valvular Intercellular Communication

**DOI:** 10.3389/fmolb.2021.749681

**Published:** 2021-11-05

**Authors:** Benedikt Bartsch, Philip Roger Goody, Mohammed Rabiul Hosen, Denise Nehl, Neda Mohammadi, Andreas Zietzer, Philip Düsing, Alexander Pfeifer, Georg Nickenig, Felix Jansen

**Affiliations:** ^1^ Department of Internal Medicine II, Heart Center Bonn, University Hospital Bonn, Bonn, Germany; ^2^ Institute of Pharmacology and Toxicology, University Hospital Bonn, Bonn, Germany

**Keywords:** ncRNA, atherosclerosis, aortic stenosis, endothelial dysfunction, ncRNA therapy

## Abstract

Non-coding RNAs have been shown to be important biomarkers and mediators of many different disease entities, including cardiovascular (CV) diseases like atherosclerosis, aneurysms, and valvulopathies. Growing evidence suggests a central role of ncRNAs as regulators of different pathological pathways involved in endothelial dysfunction, cardiovascular inflammation, cell differentiation, and calcification. This review will discuss the role of protein-bound and extracellular vesicular-bound ncRNAs as biomarkers of vascular and valvular diseases, their role as intercellular communicators, and regulators of disease pathways and also highlights possible treatment strategies.

## Introduction

Non-coding RNAs (ncRNAs) consists of transfer RNA (tRNA), microRNA (miRNA, or miR), long noncoding RNA (lncRNA), circular RNA (circRNA), and other small RNAs. NcRNA expression has been shown to correlate with several cardiovascular diseases including aortic stenosis. Modulating ncRNA expression *in vitro* has also been shown to affect disease progression (Das, 2020). While considerable advances in understanding the molecular functions of ncRNAs *in vitro* have been achieved in the last years, unravelling the role of ncRNAs *in vivo*, their establishment as biomarkers and possible use as potential therapeutics are still in its infancy. Therefore, ncRNAs are promising targets for further research.

## Regulation of Cellular NcRNA Expression Under Physiological and Pathological Conditions

NcRNA expression in CV cells can vary, depending on the pathophysiological condition of the parent cell. Different stimuli, such as glucose levels, oxidative stress, inflammation, and an osteogenic milieu—all important in CV pathologies—can influence ncRNA expression in the affected cells (Libby et al., 2019; Yuan et al., 2019). Current knowledge on synthesis and maturation of different classes of ncRNAs, under physiological and pathological conditions, will be summarized and discussed in this section.

### Hyperglycemia

High plasma glucose concentrations are a major risk factor for atherosclerosis as well as aortic stenosis (Cosentino, 2019; Goody et al., 2020). *In vitro*, human umbilical vascular endothelial cells (HUVECs) exposed to high glucose medium displayed an upregulation of 214 lncRNAs, while 197 were downregulated and several ncRNAs interfered directly with glucose metabolism, while 945 possible lncRNA-mRNA pairs were found, indicating a strong regulatory link (Sun and Wong, 2016; Xu et al., 2020a).

In a diabetic mouse model, the lncRNA metastasis associated lung adenocarcinoma transcript 1 (MALAT1) mediated pro-inflammatory cytokine expression was altered according to glucose concentration and MALAT1 inhibition lead to a diminished inflammatory response as well as reduced endothelial cell apoptosis and tube formation in retinal cells (Radhakrishnan and Kowluru, 2021).

Plasmacytoma variant translocation 1 (PVT1), another apoptosis mediator, is upregulated in kidney cells exposed to high glucose levels and mediates hypoxic cardiac injury by acting as a sponge for miR-135a-5p, thus upregulating Forkhead box O1 (FOXO1)-mediated apoptosis (Sun and Wong, 2016; Xu et al., 2020b).

Recently, Liu et al. demonstrated a glucose-dependent steroid receptor RNA activator (SRA) mediated increase in insulin sensitivity, most likely via the insulin-like-growth-factor 1 (IGF1) and PPARγ signaling pathway (Liu et al., 2014a; Liu et al., 2014b; Liu et al., 2016). Unlike IGF1 and PPARγ, SRA co-activation negatively regulates Toll-like-recptor 4 (Tlr4) and subsequent TNFα release, both of which have been linked to the pro-inflammatory response in the early stages of atherosclerosis and aortic stenosis (Chong et al., 2004; Xu et al., 2010; Liu et al., 2016; Goody et al., 2020).

The lncRNA myocardial infarction-associated transcript (MIAT) has initially been associated with myocardial infarction but can also act as a sponge for miR-150-5p, which regulates VEGF-expression, and its expression is increased in patients with renal dysfunction and high blood glucose levels (Yan et al., 2015; Sun and Wong, 2016).

### Oxidative Stress

Another mediator of vascular and valvular damage on a cellular level is oxidative stress (Kattoor et al., 2017; Goody et al., 2020).

NcRNAs are differentially expressed in monocytes and macrophages isolated from human blood samples from patients with and without high risk for atherosclerosis (Liu et al., 2014c; Yan et al., 2015). Linc-TP53I13 and linc-POTED8 are overexpressed in an *in vitro* model mimicking oxidative stress through lipopolysaccharide exposure in monocytes and adipocytes and in obese patients (Liu et al., 2014c). MIAT1 expression is increased in cells exposed to oxidative stress (Yan et al., 2015).

### Inflammation

TNFα is major signaling molecule in innate and adaptive immunity responses in different tissues (Whitley et al., 1994). One of its many pro-inflammatory downstream signaling pathways includes the NF-κB pathway, which induces gene expression of cytokines such as IL-1, and different miRs as well as lncRNA (e.g., LincRNA-Cox2). The regulated ncRNAs have been shown to often lie adjacent to coding genes that were also regulated by NF-κB such as Cox2 Divergent and Gp96 Convergent (Rapicavoli et al., 2013).

LincRNA-Cox2 is found proximally to the prostaglandin-endoperoxide synthase 2 (Cox2) gene locus and its expression is promoted in a pro-inflammatory environment, such as after TLR-2 and -4 stimulation or after LPS stimulation in macrophages *in vitro* (Guttman et al., 2009; Carpenter et al., 2013). The activation of TLRs plays a key role in atherosclerosis and myocardial infarction (Chong et al., 2004). LincRNA-Cox2 downregulates the expression of immune genes, similar to an auto-feedback-mechanism, by binding to heterogeneous nuclear ribonucleoproteins (hnRNPs) in order to repress transcription (Carpenter et al., 2013).

**FIGURE 1:**
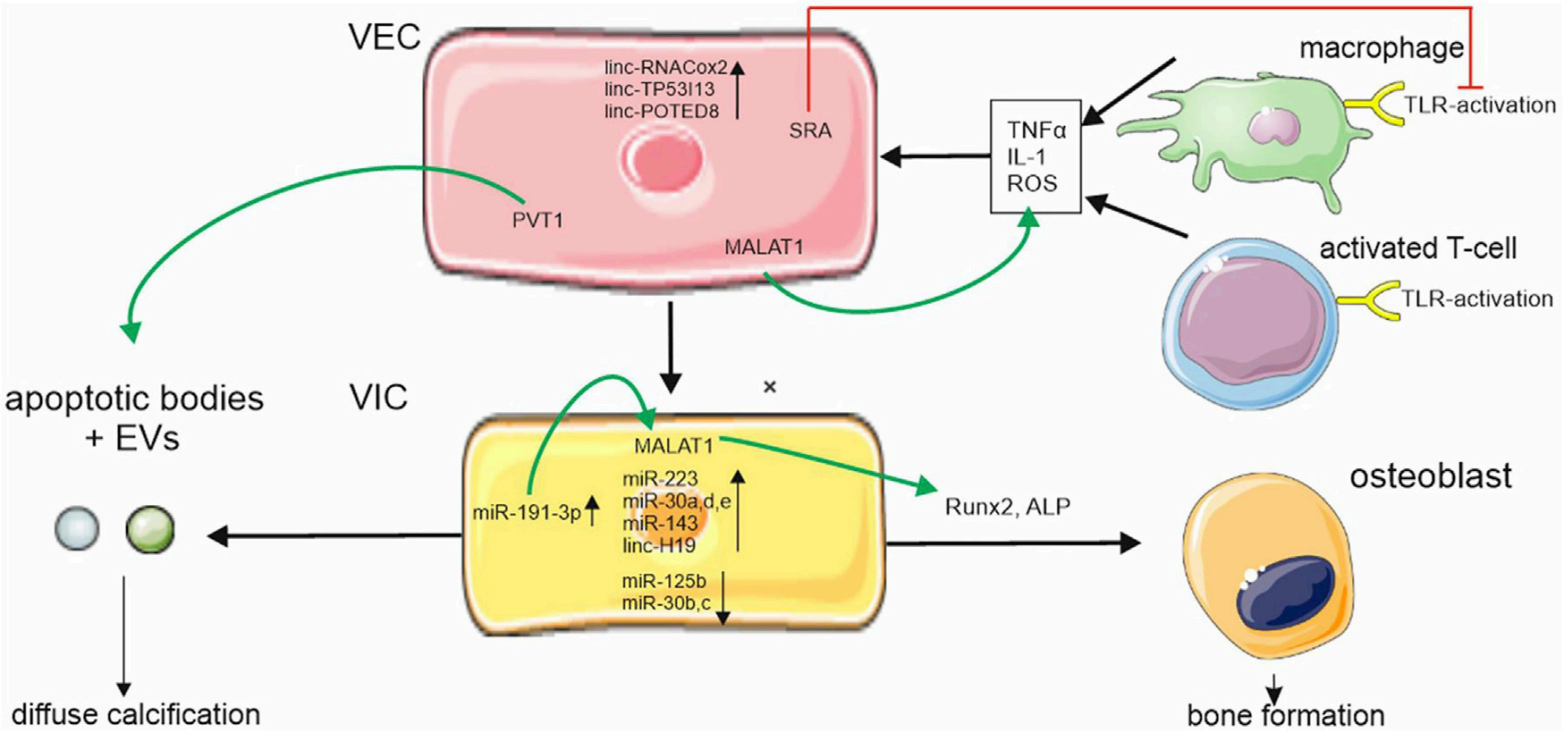
ncRNA regulation in aortic stenosis. ncRNAs are differently expressed during aortic stenosis disease progression in valvular endothelial cells (VEC) and valvular interstitial cells (VIC). Metastasis associated lung adenocarcinoma transcript 1 (MALAT1) is upregulated in VECs und VICs and increases pro-inflammatory cytokine expression and regulators of osteoblastic differentiation. Plasmacytoma variant translocation (PVT1) regulates apoptosis. Apoptosis is part of a complex system leading to programmed cell death thus causing increased calcium uptake of VICs through cell debris steroid receptor RNA activator (SRA) which downregulates Toll-like receptor (TLR) activation. Cellular symbols were adopted with permission from smart.servier.com and Vecteezy.

While ncRNAs were shown to regulate inflammatory responses in a variety of cardiovascular diseases, the number of studies investigating the role of ncRNAs in aortic stenosis remains low. Yet, key promoters of aortic stenosis such as TNFα, members of the Wnt-pathway, and TLR activation are modulated by ncRNA expression and thus may provide a promising target for future investigations (see Figure 1) (Wang et al., 2013; Venardos et al., 2014; Goody et al., 2020). Recently published data suggests a key role of ncRNA in regulating oxLDL-uptake, endothelial-to-mesenchymal-transformation (EndMT) and valvular calcification (Mahmut et al., 2014; Rayner, 2020).

### Osteogenic Milieu

NcRNA expression is also modified in osteogenic milieus and can promote osteoblastic differentiation of cardiovascular cells, a major driver of calcific valve disease and atherosclerotic plaque development (Alexopoulos and Raggi, 2009; Goody et al., 2020). MiR-30 family members moderate mesenchymal stem cell (MSC) transformation to osteocytes by regulating Runx2-expression (Eguchi et al., 2013). In human aortic valvular interstitial cells that have been treated with an osteogenic medium, miR-30 b/c are downregulated during MSC transformation, while miR-30 a, d, and e are upregulated with miR-30e acting as a co-activator of the Wnt-pathway and inductor of Runx2 expression in human coronary artery smooth muscle cells (HCASMC) (Nigam et al., 2010; Eguchi et al., 2013; Wang et al., 2013). MiR-125b, miR-143 and -145 are downregulated *in vitro* in human vascular smooth muscle cells (HVSCM) after treatment with high levels of inorganic phosphate to promote osteogenic differentiation in these cells (Coffey and Jones, 2014). Furthermore, miR-125b was found to be downregulated in an *in vivo* model of atherosclerosis and aortic calcification in mice (Goettsch et al., 2011; Rangrez et al., 2012; Coffey and Jones, 2014), while miR-223 was overexpressed in calcified murine aortas (Rangrez et al., 2012). Human calcific aortic valve disease is associated with significantly reduced miR-204 levels and miR-204 mimics suppressed the osteogenic activity of interstitial cells from diseased valves (Song et al., 2020).

Furthermore, MALAT1 has been demonstrated to promote osteogenic differentiation in an osteogenic milieu via an increased ALP activity and Runx2 activation (Wang et al., 2020). MALAT1 is regulated in an osteogenic milieu via human antigen R (HuR) expression, which itself is upregulated by MALAT1 via inhibition of miR-191-3p, establishing a positive feedback loop for osteogenic differentiation (Wang et al., 2020).

## Cellular Selection and Packaging Mechanisms of NcRNAs Into Extracellular Carriers

Growing evidence suggests that secreted ncRNA profile reflects the state of the parent cell and can be released directly into the extracellular space/blood stream, bound to RNA-binding proteins such as Argonaute 2 (Ago-2) or be (selectively) packaged into different forms of extracellular vesicles (EVs) (Kim et al., 2017). These EVs include exosomes, microvesicles and apoptotic bodies.

The ncRNA content of EVs often does not reflect their corresponding concentrations in the cytoplasm of the originating cell, thus underlining the concept that ncRNA sorting into EVs and secretion are actively regulated cellular processes that are relevant for intercellular communication (Villarroya-Beltri et al., 2013; Gezer et al., 2014; Shurtleff et al., 2016).

MiRNAs with gene sequences GGAG, C/UCCU/G, so called EXOmotifs, in the 3′ half of the RNA were found overrepresented in EVs, while miRNAs with mutated EXOmotifs were not detected in EVs, indicating a cause-effect relation between EXOmotifs and EV packaging, potentially mediated *via* heterogeneous nuclear ribonucleoproteins (HNRNP) (Villarroya-Beltri et al., 2013; Zietzer et al., 2020). EV sorting functions as a tool for cellular ncRNA homeostasis. Thus, ncRNA EV levels are reduced when their cellular binding proteins or target mRNAs are artificially overexpressed (Gao and Wu, 2015).

NcRNA can also be found outside of EVs in all body fluids (Tzimagiorgis et al., 2011) and their composition differs significantly between EVs and the non-EV secretome, with miRNAs composing the largest fraction of ncRNA found in both compartments (Langevin et al., 2020). Secretion of unbound ncRNA seems to be associated with necrosis and apoptosis of the releasing cell. Since EV-unbound DNA and RNA is similarly fragmented as the DNA/RNA in apoptotic bodies, unbound DNA/RNA could also originate from apoptotic bodies (Halicka et al., 2000; Li et al., 2003).

EV uptake into target cells is mediated through a variety of pathways (Maas et al., 2017). EVs can interact with their target cells via specific ligand-receptor interactions such as clathrin-mediated endocytosis and activate downstream signaling pathways (Mulcahy et al., 2014; Costa-Silva et al., 2015). In order for ncRNAs to carry out their cellular functions, EVs not only have to bind to their target cells but need to deliver their cargo into the cytoplasma of the cell, most likely via endocytosis (Mulcahy et al., 2014). Phagocytosis of EVs is promoted when their content is lipid-rich and the extracellular environment is acidic (Parolini et al., 2009; Mulcahy et al., 2014). To prevent degradation *via* lysosomal fusion, ncRNAs need to escape this compartment before fusion (Stalder et al., 2013). Uptake of EV-cargo into the endosome is regarded as a potential escape mechanism for ncRNA degradation (Maas et al., 2017). NcRNAs may avoid degradation by binding to Ago and interact with the RNA interference silencing complex (RISC), a multi-protein complex at the ER that uses ncRNA as a template to cleave the corresponding mRNA (Pratt and MacRae, 2009; Stalder et al., 2013). EVs were found to encircle the ER before fusing with lysosomes, thus potentially allowing RISC and ncRNA interaction, induction of miRNA/mRNA degradation and silencing protein translation, rather than sole miRNA degradation (Barman and Bhattacharyya, 2015).

Under ischemic conditions, cardiomyocytes transmit EVs promoting inflammation *via* IL6 and CC2 release. In contrast, endothelial cells can prevent cardiomyocyte apoptosis in ischemia *via* EV secretion and miR-transfer (Davidson et al., 2018; Loyer et al., 2018). In calcific aortic valve disease (CAVD) EVs and ncRNA expression appear to regulate the initial inflammatory phase and may promote calcification of the valve (Hutcheson et al., 2014; Bakhshian Nik et al., 2017).

In summary, ncRNA mediate a variety of cardiac diseases through uptake into endothelial cells, smooth muscle cells, cardiomyocytes as well as immune cells.

## The Non-Coding Transcriptome as Biomarker: Challenges and Future Directions

NcRNAs have become of great interest as biomarkers of various diseases (Busch et al., 2016). Circulating EV- and protein-bound ncRNAs have been shown to be differentially expressed in patients with and without CV diseases such as atherosclerosis, aortic aneurysms, aortic valve stenosis, and (pulmonary) hypertension (Liu et al., 2019). Levels of ncRNAs can be either increased or decreased and correlate with disease outcome, thus demonstrating their ability to serve as biomarkers of CV disease.

NcRNAs have been tested as screening biomarkers for several cardiovascular diseases such as myocardial infarction (MI), coronary artery disease (CAD) and heart failure. In myocardial infarction miRNA-1, -133a/b, -208a, -499 became a frequently studied group referred to as myomirs due to cardiac specific interactions with different myosin chains and quick expression response after myocardial injury (van Rooij et al., 2009; Busch et al., 2016). In a study determining the correlation of miRNAs with myocardial infarction, only the levels of miRNA-134 and miR-184 appeared to correlate with infarction, with miRNA-134 promoting proliferation of cardiac progenitor cells *in vitro* (Wu et al., 2015; Busch et al., 2016). Troponin assays were superior to circulating miRNAs in predicting myocardial infarction in patients presenting with chest pain, but some miRNAs (miR-208b) showed a high predictive value for the lifetime risk of MI (Zampetaki et al., 2012; Devaux et al., 2015).

In CAD miRNA-133a and miRNA-499 showed a positive correlation with vessel calcification, while miRNA-145 and -155 expression showed an inverse correlation with CAD-severity and progression (Busch et al., 2016). However, in a clinical setting no ncRNA was able to predict angina pectoris. The number of patients enrolled in this study was limited and, with miRNA-155 shown to alter atherosclerosis *in vivo* in a mouse model of atherosclerosis, larger cohorts may yield more reliable results in the future (Bhattachariya et al., 2015).

Research in lncRNA as biomarkers are less advanced due to difficulties in maintaining their structural integrity in bodily fluids over prolonged periods of time (Shi and Yang, 2016). Circulating levels of the ncRNA LIPCAR were found to be upregulated in heart failure patients and could predict cardiac remodeling in general with high LIPCAR levels associated with increased cardiac mortality (Kumarswamy et al., 2014). Vausort et al. identified three circulating lncRNAs (aHIF, KCNQ1OT1, and MALAT1) upregulated and one downregulated (ANRIL) in patients with myocardial infarction, but again all lncRNAs were inferior in predicting MI than conventional troponin assays (Vausort et al., 2014).

To analyze and quantify ncRNAs in exosomes and microvesicles, they must be isolated from platelet-depleted plasma (Liu et al., 2020). Isolation techniques differ between ncRNAs transported in EVs and those transported in ncRNA-protein complexes (Liu et al., 2020). A commonly used technique is differential centrifugation, although a variety of isolation methods are applied (Witwer et al., 2013). After purification of EVs, ncRNA isolation can be performed with phenol-containing reagents or phenol free assays (E et al., 2018). NcRNAs are then further analyzed using reverse transcription and quantitative PCR. Yield and purity differ immensely between different approaches as well as between research groups (E et al., 2018), and the low concentration of protein-ncRNA complexes can make quantification difficult (Gallo et al., 2012). While differential ultracentrifugation is an established method, it is time consuming and demands large sample sizes, while only producing a low recovery rate, thus making it impractical in large scale clinical settings (Liu et al., 2020). Other techniques, better suited for small sample sizes, such as spectrofluorimetry and capillary electrophoresis are more expensive and even more time consuming, and thus also not feasible in a high throughput diagnostic setting (E et al., 2018; Gallo et al., 2012). Cheaper and faster alternatives are size based methods such as ultrafiltration or hydrostatic filtration dialysis with commercial EV filter kits already established. Exosomes can also be isolated using a weight or size specific filter. However, increased mechanical sheer may break vesicles and influence results. With exosomes derived from the endocytic pathway and microvesicles formed from the plasma membrane, they express different CD-markers, making immunocapture-assays another potential route for isolation (Liu et al., 2020).

A general problem when using extracellular ncRNAs as potential biomarkers is their high dependency on sex, ethnicity and pre-analytical variabilities, as well as their altered concentrations after heparin, acetylsalicylic acid, or statin administration, making the definition of pathological threshold levels difficult (Moldovan et al., 2014; Viereck and Thum, 2017). While no international standards have been set, due to potential heparin interference blood serum obtained in the morning hours from fasting patients promises more reliable results. In order to harmonize standards a compendium of exosomal proteins and ncRNA by the International Society of Extracellular Vesicles (ISEV) has been established (Keerthikumar et al., 2016).

## NcRNAs as Potential Therapeutic Targets

Several pathomechanisms in cardiovascular disease are influenced by ncRNA, making them promising and desirable targets to influence by way of ncRNA mimics or inhibitors. NcRNA therapeutics do not induce drug resistance effects in target cells and can be modified to increase their half-life, making them almost ideal therapeutic molecules (Geary et al., 2015).

In order to modulate miR- or lncRNA concentration in cardiovascular and valvular disease, RNA-based therapeutics need to reach either target cells (e.g., valvular or vascular endothelial cells, cardiomyocytes, etc.) via the blood stream or local injection (Zietzer et al., 2021). Intracardial application of RNA therapeutics demands a more invasive application pathway while i.v. or s.c. application leads to systemic distribution (Ito et al., 2009). Due to limited clinical trials, it is currently unknown how and if the local intracardial concentration of miRNA therapeutics differs between different application methods (Huang et al., 2020). In murine and porcine models, intracardial antimiR-132 concentrations in cardiomyocytes showed no difference after intravenous or intracoronary injection (Foinquinos et al., 2020).

Another hurdle for ncRNA therapeutics is identifying a transporter that can deliver its cargo specifically to its target. Virus-based approaches, using a modified adeno associated virus-capsule with an increased cardiac target specificity, are seen as a reliable transport mechanism with limited systemic effects demonstrated in rhesus macaques (Mingozzi et al., 2013). However, AAV-delivery may be limited due to potentially high adenovirus antibody titers in the general population (Calcedo et al., 2011). Also, due to its small genome size (3–4 kb capacity) the inserted ncRNA size is limited, making the overexpression of lncRNAs difficult or impossible, at least with AAV-based vectors (Smith et al., 2009). AAV transfection, when successful, leads to long-term persistence, thus pro proliferative effects must be regulated via the promoter region to avoid cancerogenic transformation (Braga et al., 2021).

Alternatively, delivery methods based on EVs and liposomes are already being tested in clinical settings (Braga et al., 2021). So called lipoplexes consist of small lipid molecules and have successfully been used to transfect cardiomyocytes *in vitro* and *in vivo* in different animals and are currently tested in clinical trials (Kulkarni et al., 2018). For systemic application it is important that lipoplexes are not positively charged to avoid increased plasma clearance as well as systemic inflammation and must not be too large (< 1 µm) to avoid systemic inflammatory responses and toxicity. Only recently, smaller (< 100 nm) and ionizable or neutral lipid nanoparticles were introduced to avoid inflammation and toxicity (Kulkarni et al., 2018). For amyloidosis Patisiran is already used in a clinical setting (Hoy, 2018). Modified exosomes, through engineering of specific ligands onto the exosomal membrane, have also been tested as ncRNA transporters as well (Mathiyalagan and Sahoo, 2017). While exosome-based therapy is still in its infancy, the use of cellular organelles promises low systemic toxicity and antigenicity (Mathiyalagan and Sahoo, 2017; Braga et al., 2021). For example, trial NCT04327635 investigates patient safety in intracoronary exosomes application after myocardial infarction, which may limit systemic toxicity (McLeod, 2021).

Synthetic Nanoparticles with diameters ranging between 50 and 100 nm showed an optimized distribution of RNA therapeutics *in vivo* as well as an increased half-life (Boca et al., 2020). Their surface can easily be modified with aptamers, antibodies or peptides, potentially making them tissue specific and reducing off-target side effects (Di Mauro et al., 2018). However, their cargo capacity is limited and their effectiveness in cardiovascular cells still needs to be demonstrated (Di Mauro et al., 2018). While ncRNAs are promising targets for pharmaceutical therapy and ncRNA derived pharmaceuticals may yield almost ideal pharmacokinetic properties, their delivery method as well as their method of transportation within the body still pose major challenges.

## Conclusion

NcRNAs are important mediators in cardiovascular and valvular disease. NcRNA expression is altered according to the parental cells’ patho/physiological condition. Some ncRNAs mediate are involved in cardiovascular and valvular disease progression, while others may serve as biomarkers. EVs, lipoplexes or proteins play a key role for ncRNA transportation. Defining pathological thresholds for ncRNAs remains non-unified with ncRNA isolation techniques varying internationally. While ncRNA-based therapeutics may significantly alter cardiovascular and valvular disease progression, neither the application method nor the mode of transportation has been reliably established and this will be an important focus of future research.
